# Pathophysiology of human hearing loss associated with variants in myosins

**DOI:** 10.3389/fphys.2024.1374901

**Published:** 2024-03-18

**Authors:** Takushi Miyoshi, Inna A. Belyantseva, Mrudhula Sajeevadathan, Thomas B. Friedman

**Affiliations:** ^1^ Laboratory of Molecular Genetics, National Institute on Deafness and Other Communication Disorders, National Institutes of Health, Bethesda, MD, United States; ^2^ Division of Molecular and Integrative Physiology, Department of Biomedical Sciences, Southern Illinois University School of Medicine, Carbondale, IL, United States

**Keywords:** hearing, myosin, stereocilia, cargo transport, hereditary deafness

## Abstract

Deleterious variants of more than one hundred genes are associated with hearing loss including *MYO3A*, *MYO6*, *MYO7A* and *MYO15A* and two conventional myosins *MYH9* and *MYH14*. Variants of *MYO7A* also manifest as Usher syndrome associated with dysfunction of the retina and vestibule as well as hearing loss. While the functions of MYH9 and MYH14 in the inner ear are debated, MYO3A, MYO6, MYO7A and MYO15A are expressed in inner ear hair cells along with class-I myosin MYO1C and are essential for developing and maintaining functional stereocilia on the apical surface of hair cells. Stereocilia are large, cylindrical, actin-rich protrusions functioning as biological mechanosensors to detect sound, acceleration and posture. The rigidity of stereocilia is sustained by highly crosslinked unidirectionally-oriented F-actin, which also provides a scaffold for various proteins including unconventional myosins and their cargo. Typical myosin molecules consist of an ATPase head motor domain to transmit forces to F-actin, a neck containing IQ-motifs that bind regulatory light chains and a tail region with motifs recognizing partners. Instead of long coiled-coil domains characterizing conventional myosins, the tails of unconventional myosins have various motifs to anchor or transport proteins and phospholipids along the F-actin core of a stereocilium. For these myosins, decades of studies have elucidated their biochemical properties, interacting partners in hair cells and variants associated with hearing loss. However, less is known about how myosins traffic in a stereocilium using their motor function, and how each variant correlates with a clinical condition including the severity and onset of hearing loss, mode of inheritance and presence of symptoms other than hearing loss. Here, we cover the domain structures and functions of myosins associated with hearing loss together with advances, open questions about trafficking of myosins in stereocilia and correlations between hundreds of variants in myosins annotated in ClinVar and the corresponding deafness phenotypes.

## 1 Introduction

**FIGURE 1:**
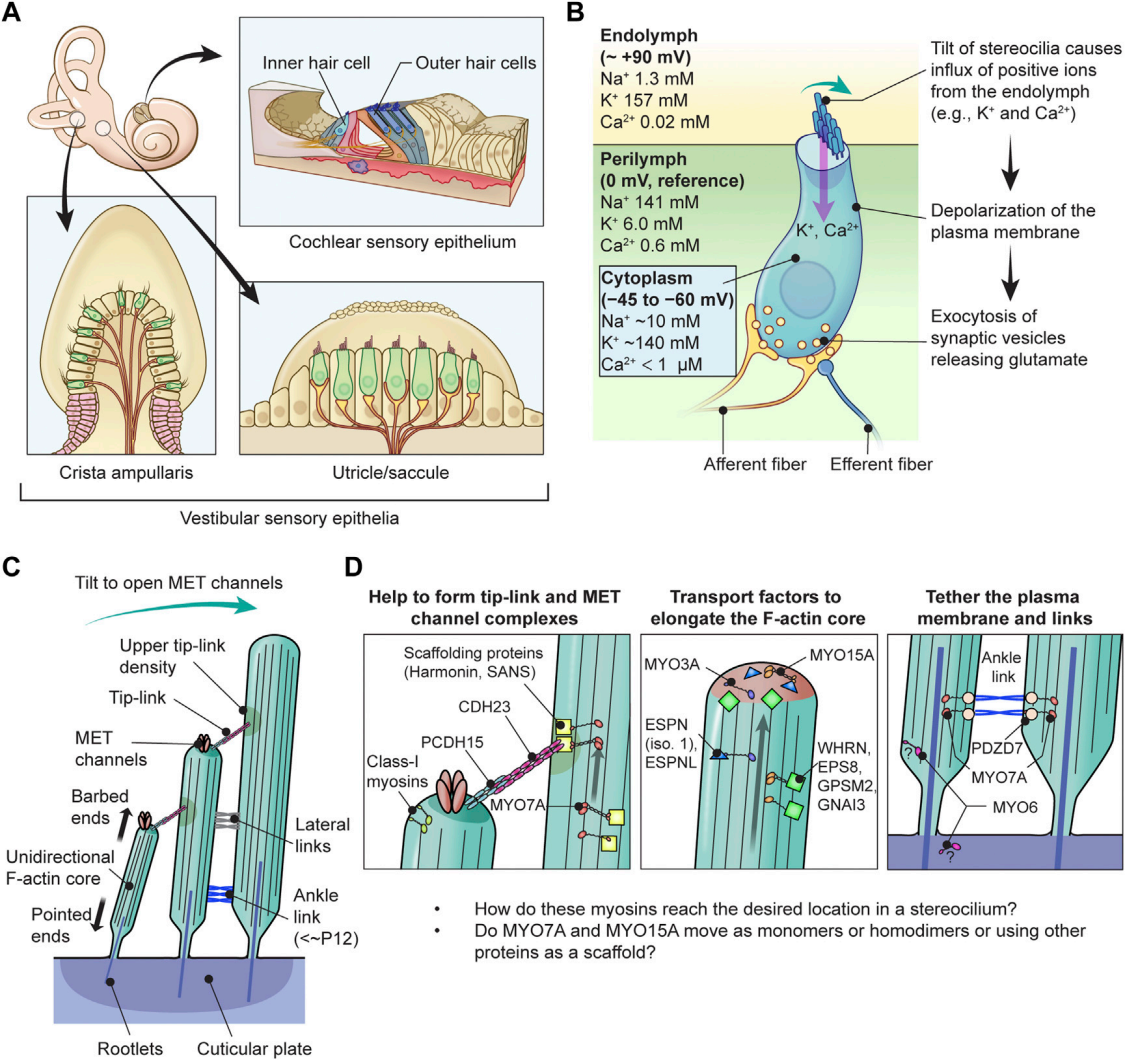
Functions of myosins in stereocilia of inner ear hair cells **(A)** Sensory epithelia in the inner ear. The cochlear sensory epithelium forms a spiral and has one row of IHCs and three rows of OHCs to detect sound stimuli (top right). Vestibular sensory epithelia are in the utricle, the saccule and three cristae ampullaris. Epithelia in the utricle and saccule detect linear acceleration in different orientations and gravity (bottom right). Epithelia in the cristae ampullaris cover angular accelerations in three-dimensional space using the fluid motion in semicircular canals perpendicular to each other (bottom left). **(B)** Inner hair cell schematically showing the mechanotransduction event during sound stimulation. Ion concentrations are previously reviewed (Wangemann, 2006; McPherson, 2018). The plasma membrane of cochlear hair cells is negatively charged between −45 mV and −60 mV against the perilymph, which have a gradient along the cochlear length (Purves, 2018), and has a steeper gradient against the positively charged endolymph, which is approximately +90 mV against the perilymph (Li et al., 2020). Deflection of stereocilia allows positive K^+^ and Ca^2+^ ions in the endolymph to enter the cell body through MET channels at stereocilia tips and depolarizes the plasma membrane from the resting potential. This depolarization triggers synaptic vesicle release at the base of hair cell initiating signal transmission through the afferent fiber synapses (yellow) to the cochlear nerve. Regulatory efferent fiber endings (dark blue) are connected to the afferent nerve endings in sound transducing mature inner hair cells. **(C)** Architecture of stereocilia. The MET channels are localized at the tips of stereocilia and connected by tip-links to the side of adjacent stereocilia of the longer row, which is referred to as the upper tip-link density (UTLD) based on the high scattering of electrons in transmission electron microscopy. Each stereocilium contains a core of tightly-packed unidirectional F-actin which narrows down and is connected to the cuticular plate. Connection between the F-actin core and the cuticular plate is supported by rootlets consisting of more tightly packed F-actin. **(D)** Major functions of myosins in a stereocilium. MYO7A is localized at the UTLD and involved in localization of components of the tip-link and the MET channel (and also formation of ankle-links, right panel). Some class-I myosins are reported to play a role in adaptation during sustained sound stimulation. MYO3A and MYO15A accumulate at stereocilia tips and both transport cargo essential for elongating the F-actin core. MYO6 is thought to connect the plasma membrane to the F-actin core at stereocilia tapered base and also function in the cuticular plate.

Sound waves pass through the outer and middle ear providing input signals to the mechanosensory organelles on the apical surface of cochlear hair cells. These organelles represented by cylindrical protrusions called stereocilia are derived during development from microvilli and are aligned in rows of graded heights that synchronously deflect in response to sound vibrations in inner ear fluids (Schwander et al., 2010) (Figures 1A–C). Mechanical sound stimuli are converted by stereocilia into electro-chemical activities in hair cells. With the exception of the tallest row, stereocilia are equipped with mechanotransduction (MET) channels at their distal ends, which are connected to the side of adjacent longer stereocilia by tip-links that gate the MET channels (Gillespie and Muller, 2009). Open MET channels allow cations in the endolymph, specifically K^+^ and Ca^2+^, to flow into the hair cell cytoplasm. This influx causes a depolarization wave of the plasma membrane that propagates toward the base of hair cells, induces the opening of voltage-dependent Ca^2+^ channels and finally triggers Ca^2+^-dependent fusion of glutamate-containing synaptic vesicles at the basal surface of hair cells (Corey and Hudspeth, 1979; 1983; Glowatzki and Fuchs, 2002) (Figure 1B).

Each stereocilium has a core of unidirectional F-actin tightly packed by actin cross-linkers, including ESPN, PLS1, FSCN2 and XIRP2 (Zheng et al., 2000; Shin et al., 2010; Scheffer et al., 2015; Krey et al., 2016), and connected to the underneath cuticular plate by a rootlet consisting of F-actin bundled more tightly by TRIOBP4 and TRIOBP5 (Kitajiri et al., 2010; Katsuno et al., 2019) (Figure 1C). For stereocilia, the F-actin core is a backbone to resist repeated mechanical stimuli and also a scaffold for various proteins including components of the MET machinery and motor proteins to transport or anchor these components. A similar mechanism is utilized by vestibular hair cells to detect gravity and acceleration as inputs for equilibrioception (Eatock and Lysakowski, 2006).

The myosin superfamily is one of three major families of motor proteins along with the kinesins and dyneins. Only myosins move on F-actin (Sweeney and Holzbaur, 2018). A typical myosin molecule has an ATPase head motor domain to transmit forces to F-actin, a neck containing IQ-motifs that bind regulatory light chains and a tail region to interact with specific partners. Phylogenic analyses have revealed 18 classes of myosins (Foth et al., 2006) including class-II “conventional” double-headed myosins initially discovered in skeletal muscle extracts (Kühne, 1864) and other “unconventional” myosins named after the single-headed class-I myosin discovered in *Acanthamoeba* (Pollard and Korn, 1973). While the tails of class-II myosins consist of a long coiled-coil domain to form a double-headed homodimer, those of unconventional myosins contain various motifs to recognize specific partners and usually have no or only a short coiled-coil domain (Batters and Veigel, 2016). Myosins lacking the coiled-coil domain are “unconventionally” single-headed because they cannot dimerize spontaneously. As another difference from conventional myosins, some unconventional myosins can “walk” on F-actin when dimerized by a short coiled-coil domain (e.g., class V and X) or by cargo bound to the tail (e.g., class VI and VIIA) and can transport vesicles and specific proteins as cargo (Yu et al., 2009; Sakai et al., 2011; Batters and Veigel, 2016; Liu et al., 2021). In the human genome, there are about 40 different genes encoding myosins that are grouped into 12 classes, I–III, V–VII, IX, X, XV, XVI, XVIII and XIX (Foth et al., 2006). Abnormal function of these myosins are associated with various diseases including myopathies, colitis, glomerulosclerosis, neurological defects, cancer, blindness and hearing loss (Coluccio, 2020).

In humans, variants of two genes encoding class-II conventional myosins, *MYH9* and *MYH14*, and four genes encoding class-IIIA, -VI, -VIIA and -XVA unconventional myosins, *MYO3A*, *MYO6*, *MYO7A* and *MYO15A,* are presently associated with autosomal dominant or recessive nonsyndromic sensorineural hearing loss (Table 1). Variants of *MYO7A* are also associated with autosomal recessive Usher syndrome type 1 characterized by congenital sensorineural hearing loss, vestibular dysfunction and progressive retinitis pigmentosa (Weil et al., 1995). While the functions of MYH9 and MYH14 in the inner ear are still being debated, MYO3A, MYO6, MYO7A, MYO15A and some class-I myosins (e.g., MYO1C) are expressed in inner ear hair cells and are crucial for developing and maintaining functional stereocilia by anchoring and/or transporting specific partners on the F-actin core (Table 2). Decades of studies have identified the cargo of these unconventional myosins in a stereocilium and elucidated their contribution to normal hearing including (1) formation of the MET machinery, (2) elongation of the F-actin core and (3) tethering the plasma membrane and the ankle links to the F-actin core (Friedman et al., 2020) (Figure 1D). However, less is understood about how these myosins traffic in a stereocilium using their motor function. For example, it is unknown whether or not MYO7A functions as a dimer (or perhaps as an oligomer) in a stereocilium although cargo-mediated dimerization is reported for this myosin and likely utilized in the retina (El-Amraoui et al., 2002; Sakai et al., 2011).

**TABLE 1:** Myosins associated (or potentially associated) with human hearing loss and their kinetic properties. Prepared based on data published in reviews (O'Connell et al., 2007; Heissler and Manstein, 2013). The italic values indicate gene names.

Gene	Locus	Duty ratio	Rate-limiting step	Velocity (µm/s) ^†^	References
*MYO1A*	Refuted	0.05	Pi release	0.07–0.1 at 37°C	Collins et al. (1990), Ostap and Pollard (1996), Jontes et al. (1997), Eisenberger et al. (2014), Patton et al. (2016)
*MYO1C*	Disputed	0.11	Pi release	∼0.06 at 37°C	Stauffer et al. (2005), Lewis et al. (2006), Lin et al. (2011), Greenberg et al. (2012), DiStefano et al. (2019)
*MYO1F*	Disputed	N.A.	N.A.	N.A.	DiStefano et al. (2019)
*MYO3A*	*DFNB30*	0.25 (0.91^‡^)	ADP release	0.11 at RT	Walsh et al. (2002), Komaba et al. (2003), Dose et al. (2007), Salles et al. (2009), Cirilo et al. (2021)
*MYO6*	*DFNA22*, *DFNB37*	0.8 (monomer)	ADP release	−0.131 (monomer), −0.307 (dimer) at 30°C^§^	Avraham et al. (1997), Self et al. (1999), De La Cruz et al. (2001), Melchionda et al. (2001), Ahmed et al. (2003a), Goodyear et al. (2003), Morris et al. (2003), Sakaguchi et al. (2008)
*MYO7A*	*DFNA11*, *DFNB2*, *USH1B*	0.9 (monomer)	ADP release	0.19 at RT	Weil et al. (1995), Liu et al. (1997), Weil et al. (1997), Udovichenko et al. (2002), Watanabe et al. (2006), Lefevre et al. (2008), Grillet et al. (2009), Dionne et al. (2018), Jaiganesh et al. (2018)
*MYO15A*	*DFNB3*	∼0.5	ATP binding	∼0.43 at 30°C	Wang et al. (1998), Beyer et al. (2000), Mogensen et al. (2007), Zampini et al. (2011), Bird et al. (2014), Jiang et al. (2020), Moreland et al. (2021)
*MYH9*	*DFNA17*	∼0.29	Attachment to actin or Pi release^¤^	0.29 at 30°C	Lalwani et al. (2000), Wang et al. (2000), Yengo et al. (2012)
*MYH14*	*DFNA4A*	∼0.34	Attachment to actin or Pi release^¤^	0.090 at 30°C	Donaudy et al. (2004), Heissler and Manstein (2011), Yengo et al. (2012)
*(MYO5A)*	*	0.7 (monomer)	ADP release	0.311 at 23°C	De La Cruz et al. (1999), Mehta et al. (1999)
*(MYO10)*	*	0.16, 0.6 ^¶^	N.A.	0.17 at 25°C^€^	Homma et al. (2001), Homma and Ikebe (2005), Kovacs et al. (2005)

*Listed for comparison.

†F-actin sliding assay.

‡contains the N-terminal kinase domain.

§Moves toward the pointed end of F-actin.

^¤^In solution as discussed in a comparative study (Yengo et al., 2012).

¶Another study supports the high duty ratio of MYO10 (Takagi et al., 2014).

^€^(Homma et al., 2001; Homma and Ikebe, 2005).

**TABLE 2:** Presumed functions, N-terminal splicing variants and major phenotypes in mouse models.

Myosin	Presumed functions in the inner ear	N-terminal splicing variants	Major phenotypes in knockout mice	References
MYO1A	Unclear	Not found yet	Knockout mice show no overt phenotypes	Stauffer et al. (2005), Tyska et al. (2005), Solanki et al. (2021)
MYO1C	Adaptation of MET channels	Not found yet	Homozygous knockout is associated with visual impairment. Hearing loss has not been reported in peer-reviewed journals	Stauffer et al. (2005), Solanki et al. (2021)
MYO1F	Unclear	Not found yet	Knockout mice show impaired immune responses, but hearing loss is not reported	Kim et al. (2006)
MYO3A	Transports factors to elongate the F-actin core	Not found yet	Double knockout of *Myo3a* and *Myo3b* results in profound deafness and a dysmorphic staircase architecture of stereocilia	Salles et al. (2009), Lelli et al. (2016)
MYO6	Tethers plasma membrane to the F-actin core, keeps stereocilia in place and mediates vesicle transport including endocytosis	Not found yet	Loss of MYO6 function results in profound hearing loss and stereocilia bifurcated or fused with each other	Avraham et al. (1995), Avraham et al. (1997), Self et al. (1999), De La Cruz et al. (2001), Melchionda et al. (2001), Ahmed et al. (2003a), Goodyear et al. (2003), Morris et al. (2003), Sakaguchi et al. (2008)
MYO7A	Helps to form tip-link and MET channel complexes and to tether ankle links during development	MYO7A-C, MYO7A-S	Mice lacking the MYO7A function (e.g., *Shaker-1* mice) develop severely deformed stereocilia and show profound hearing loss	Weil et al. (1995), Liu et al. (1997), Weil et al. (1997), Self et al. (1998), Udovichenko et al. (2002), Watanabe et al. (2006), Lefevre et al. (2008), Grillet et al. (2009), Dionne et al. (2018), Jaiganesh et al. (2018)
MYO15A	Transport factors to elongate the F-actin core, nucleates actin monomers and maintains the length of mechanotransducing stereocilia	MYO15A-1, MYO15A-2, MYO15A-3	Mice lacking the MYO15A function (e.g., *Shaker-2* mice) show short stereocilia and profound hearing loss	Wang et al. (1998), Beyer et al. (2000), Belyantseva et al. (2003), Belyantseva et al. (2005), Mogensen et al. (2007), Zampini et al. (2011), Bird et al. (2014), Fang et al. (2015), Jiang et al. (2020), Moreland et al. (2021)
MYH9	Unclear	Not found yet	*Myh9*-null mice are embryonic lethal. Mice with mutant *Myh9* show a phenotype resembling human MYH9-related disease including platelet dysfunction and mild hearing loss	Lalwani et al. (2000), Wang et al. (2000), Mhatre et al. (2007), Yengo et al. (2012), Zhang et al. (2012)
MYH14	Unclear	Not found yet	*Myh14*-null mice are susceptible to noise-induced hearing loss	Donaudy et al. (2004), Heissler and Manstein (2011), Yengo et al. (2012), Fu et al. (2016)

Variants of these myosin genes affecting the coding regions can result in sensorineural hearing loss through amino-acid sequences altered by nonsense, frameshift and missense mutations and also by in-frame deletions and insertions. Variants in noncoding regions can also result in hearing loss by damaging splicing of the primary mRNA transcript or by altering its expression levels (Anna and Monika, 2018; Walavalkar and Notani, 2020). Detecting and validating putative regulatory variants of a human gene associated with a disorder is challenging (Zhang and Lupski, 2015). One open question in the hearing research field is how a specific variant in these myosin genes correlates with the mode of inheritance and the severity of a clinical phenotype. For example, variants of *MYO7A* are associated with both autosomal dominant (DFNA) and recessive (DFNB) nonsyndromic hearing loss as well as Usher syndrome type 1 (USH) (Weil et al., 1995; Liu et al., 1997; Weil et al., 1997). Phenotypes are different among the *MYO7A* variants associated with autosomal DFNB hearing loss in severity and threshold at each frequency and also in the age of onset (Tamagawa et al., 2002; Riazuddin et al., 2008; Schultz et al., 2011). One approach to clarify the phenotypic spectrum of variants is to use databases, such as ClinVar (https://www.ncbi.nlm.nih.gov/clinvar/), the Deafness Variation Database (https://deafnessvariationdatabase.org/) and gnomAD (https://gnomad.broadinstitute.org/), which comprehensively curate variants with information including pathogenicity and clinical outcomes. Such databases can even point to a “hotspot” of variants in a domain whose function is not fully understood. For example, see *MYO15A* variants in Figure 6C. In this review, using ClinVar as a source of curated variants of myosin genes associated with human hearing loss, we describe the association between various myosins and hearing loss including recent advances in protein structure and function. Also discussed are open questions about trafficking of myosins in a stereocilium.

## 2 Domain structure and kinetic properties of myosins

**FIGURE 2:**
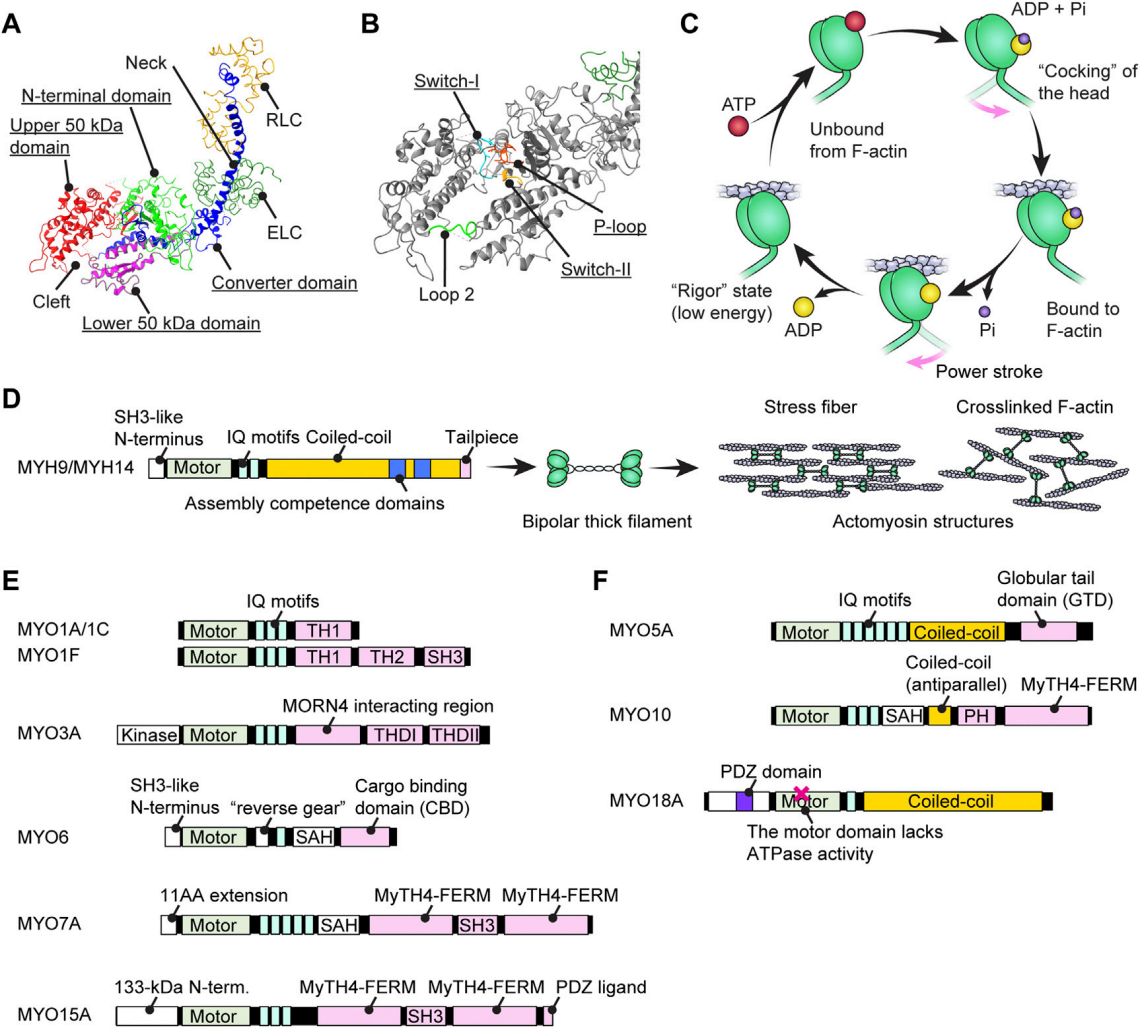
Structure of myosins and associations with human hearing loss **(A)** Four (sub)domains of the motor domain (underlined) illustrated using the structure of myosin subfragment-1 from *Gallus gallus* (PDB: 2MYS) (Rayment et al., 1993). There is a large cleft between the upper and lower 50-kDa domains. Essential and regulatory light chains are also included. **(B)** Magnified image of the motor domain in **(A)** showing three amino-acid loops crucial for ATP hydrolysis (underlined). Loop 2 connects the upper and lower 50-kDa domains. **(C)** Schematic cycle of ATP hydrolysis illustrated based on previous reviews (Houdusse and Sweeney, 2016; Sweeney and Holzbaur, 2018). Conformational changes causing F-actin binding, unbound from F-actin and a power stroke are tightly linked to each step of ATP hydrolysis. **(D)** Domain structure of conventional myosins associated with human hearing loss. MYH9 and MYH14 have a long coiled-coil to dimerize and then form actomyosin structures. The assembly competence domains are necessary for bundling in an antiparallel manner and forming a bipolar thick filament. **(E)** Domain structure of unconventional myosins associated (or potentially associated) with human hearing loss. Each myosin has unique motifs in the tail. MYO6 has a unique insertion of 53 residues called a “reverse gear” between the motor domain and the neck allowing MYO6 to move toward the pointed end of F-actin (Preller and Manstein, 2017). **(F)** Domain structure of unconventional myosins with a coiled-coil shown for comparison with **(E)**. These myosins can walk independently as a parallel dimer (MYO5A) or an antiparallel dimer (MYO10) or form a filament resembling conventional myosins (MYO18A). MYO18A has a motor domain that lacks the ATPase activity (Taft and Latham, 2020).

A myosin monomer consists of an ATPase motor domain, a neck region that binds regulatory light chains and a tail region that interacts with specific partners. The motor domain consists of four subdomains, the N-terminal domain, the upper 50-kDa domain, the lower 50-kDa domain and the converter domain as previously reviewed (Mermall et al., 1998) (Figure 2A). The N-terminal domain of class-II and class-VI myosins have SRC Homology 3 (SH3)-like β-barrel domains (Dominguez et al., 1998; Fujita-Becker et al., 2006; Niu et al., 2024), which are also reported for class-V, -XI, -XXII and -XXIV myosins (https://www.ebi.ac.uk/interpro/entry/InterPro/IPR004009/). The SH3-like domains of class-II myosins interact with the N-terminal extension of ELC (Lowey et al., 2007) while those of class-VI myosins interact with their own tails to take on a backfolded autoinhibitory state (Niu et al., 2024). The cleft between the upper and lower 50-kDa domains acts as an interface with F-actin during a stroke and is the active site for ATP hydrolysis (Rayment et al., 1996) mediated by three highly-conserved loops, the phosphate binding loop (P-loop), Switch-1 and Switch-2 (Houdusse and Sweeney, 2016) (Figure 2B). Switch-2 undergoes a large conformational change to open and close the “back-door exit” to release the inorganic phosphate (Pi) from the cleft (Yount et al., 1995). The converter domain contains an α-helix that forms a lever arm with a long helix in the neck (Sweeney and Houdusse, 2010) and amplifies the small conformational change at the active site into a large stroke (Preller and Manstein, 2013).

To transmit force to F-actin, myosin repeats a cycle of hydrolyzing ATP to ADP + Pi accompanying the conformational change of the motor domain (Bagshaw and Trentham, 1974) (Figure 2C). In addition to the speed of the ATPase cycle, which can be roughly evaluated by the F-actin gliding assay, the duty ratio is another important parameter defined as the proportion of the ATPase cycle that the motor domain remains strongly bound to F-actin (O'Connell et al., 2007). A high duty ratio greater than ∼0.5 is considered to be a necessary, but not sufficient, requirement for a myosin to show processive movements on F-actin (O'Connell et al., 2007; Pollard and Lord, 2014). The cleft in the motor domain is a mutational “hotspot” for variants associated with human hearing loss. Missense mutations in the MYO7A and MYO15A motor domains causing human and mouse hearing loss often affect the residues facing the cleft (Sellers, 2000). An additional complexity is found in class-VI myosins, which have an insertion called the “reverse gear” between the converter and lever arm. MYO6 is the only myosin that moves toward the pointed end of F-actin (Wells et al., 1999) (see Figure 2E, MYO6).

The neck region consists of a long helix connected to the α-helix in the converter domain (Sweeney and Houdusse, 2010). The length and stability of the helix is a factor determining the step size of a myosin stroke (Sakamoto et al., 2005). The neck region contains α-helical IQ motifs with a consensus sequence, [I,L,V]QXXXRGXXX [R,K] (Bahler and Rhoads, 2002), or often noted more strictly for myosins, IQXXXRGXXXR (Cheney and Mooseker, 1992). The IQ motifs interact with members of the EF-hand calcium-binding protein family including ELC, RLC and CaM (Heissler and Sellers, 2014; Jiang et al., 2020). All heavy chains of conventional class-II myosins, including MYH9 and MYH14, have two IQ motifs, IQ1 and IQ2, which interact with ELC and RLC, respectively, at high specificity (Rayment et al., 1993; Heissler and Sellers, 2014). Unconventional myosins have up to six IQ motifs depending on the class of myosins although some classes of myosins lack IQ motifs (e.g., class XIV and XVII) or have a different number of IQ motifs within the classes (e.g., class I and XIII) (Minozzo and Rassier, 2013). Among the myosins related to the inner ear function, interaction with CaM (class IC, VI, III and VIIA) and ELC (class IC, VI and VIIA) are reported (Heissler and Sellers, 2014). Two of the three IQ motifs of mouse MYO15 interact with CaM, RLC and ELC but preferably with RLC and ELC (Bird et al., 2014). Some light chains can regulate the power transmission from the motor domain to the tail. For example, CaM bound to the fifth IQ of MYO7A “slide” toward the N-terminus in the presence of Ca^2+^ and reduce the rigidity between IQ5 and the adjacent single α-helix (SAH) (Li et al., 2017).

**FIGURE 3:**
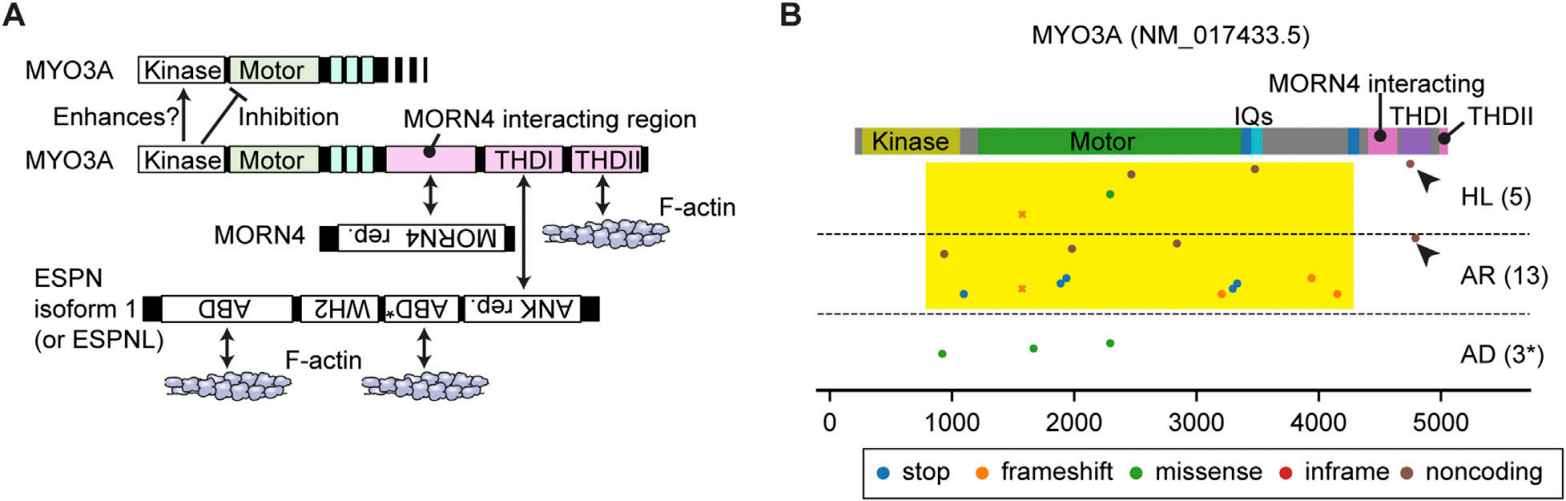
Interactome and pathogenic variants of MYO3A **(A)** Scheme showing interacting partners of MYO3A. The N-terminal kinase domain of MYO3A can phosphorylate the kinase domain and the motor domain of MYO3A (autoinhibition). The tail has region/domains that interacts with MORN4, ESPN isoform 1 (or ESPNL) and F-actin. **(B)** Pathogenic or likely pathogenic *MYO3A* variants curated by ClinVar as of 3 December 2023. Variants are plotted on the protein domains using three categories, AD: autosomal dominant nonsyndromic hearing loss, AR: autosomal recessive nonsyndromic hearing loss, HL: hearing loss with no description on the mode of inheritance. A few studies associate three variants with autosomal dominant hearing loss although the precise mechanism of the dominant inheritance is unknown (shown with asterisk). Variants in the 3′- and 5′- untranslated regions or without clinical information are not included. Most *MYO3A* variants occur in the kinase domain, the motor domain and the neck region (yellow rectangle). Two noncoding variants exist in the THDI domain that interacts with ESPN isoform 1 or ESPNL (arrowheads). Variants that belong to more than one category are plotted by crosses.

The C-terminal tail regions function as an interface for various partners including myosin itself (i.e., dimerization or multimerization) and cargo (Figures 2D–F). Class II conventional myosins MYH9 and MYH14 have a long coiled-coil tail of various lengths to dimerize with each other (Chantler et al., 2010) (Figure 2D). These heavy chain dimers can multimerize to form a bipolar thick filament as a part of contractile actomyosin structures (Craig and Woodhead, 2006; Brito and Sousa, 2020). In contrast, the tails of unconventional myosins are different between classes and even among myosins within a class and interact with different “cargo” proteins (Li and Zhang, 2020) (Figures 2E,F). Cargo of the unconventional myosins MYO7A and MYO15A include scaffolding proteins, such as harmonin, SANS and WHRN, to form a network of interactions referred to as an “interactome” (see Figure 3A; Figure 5A; Figure 6A and the next section). Compared with conventional myosins, only a few classes of unconventional myosins have a coiled-coil domain to form a filament (class XVIII) or to walk independently as a parallel dimer (class V) or as an antiparallel dimer (class X) (Batters and Veigel, 2016) (Figure 2F). Although five unconventional myosins (class V, VI, VII, X and XVIII) encoded in the human genome have a predicted coiled-coil domain in the tail on the C-terminal side of the IQ motifs (Peckham, 2011), some of these coiled-coil domains are likely to function as a single α-helix (SAH) just to extend the lever arm (Simm et al., 2017). For example, MYO6 can dimerize through their cargo, such as Dab2, bound to the C-terminal globular domain of the tail (Yu et al., 2009) and use SAH to extend the lever arm (Mukherjea et al., 2009). MYO7A can also dimerize via MYRIP in vertebrates (via M7BP in *Drosophila*) bound to the tail and likely use SAH to extend the lever arm although SAH itself retains weak dimerization activity (Sakai et al., 2011; Liu et al., 2021). In addition, tails of some myosins can inhibit the motor function. MYO5A, MYO6 and MYO7A can take a compact backfolded conformation until cargo binds to the tail to unleash motor function (Thirumurugan et al., 2006; Spink et al., 2008; Umeki et al., 2009).

A few myosins have extensions of various length at the N-terminus of the motor domain. Among the myosins associated with human hearing loss, MYO3A has a 30-kDa kinase domain at the N-terminus. In addition, some myosins have variation at the N-terminus caused by alternative splicing of the primary mRNA transcript (Figure 5B; Figure 6B). For MYO7A, presently two isoforms have been identified, the canonical isoform with an eleven amino-acid extension at the N-terminus (MYO7A-C) and a short isoform without it (MYO7A-S) (Figure 5B). There is speculation that MYO7A-C, mainly expressed in IHCs, is responsible for positioning the MET complex (Li S. et al., 2020). MYO15A also has at least three isoforms, an isoform with a large 133-kDa N-terminal domain (MYO15A-1), a short isoform without it (MYO15A-2) and an isoform with a novel 6-kDa N-terminal domain (MYO15A-3) (Belyantseva et al., 2003; Rehman et al., 2016; Moreland and Bird, 2022) (Figure 6B). As discussed in the next section, these three isoforms have different localizations and functions in hair cells. For MYO7A and MYO15A, missense variants in the N-terminal extension are associated with hearing loss demonstrating importance of this sequence for normal hearing in humans (Figure 5C; Figure 6C). In the next section, in greater detail, we review the functions of myosins associated with hearing loss and also describe pathogenic and likely pathogenic variants documented in ClinVar and discuss correlations between variants and hearing loss.

## 3 Myosins and hearing loss

### 3.1 Loci and variants

As summarized in Table 1, two genes encoding conventional myosins, *MYH9* and *MYH14*, and four genes encoding unconventional myosins, *MYO3A*, *MYO6*, *MYO7A* and *MYO15A*, are associated with nonsyndromic hereditary hearing loss in human. The kinetic properties and functions of these myosins are different from one another. *MYO3A* and *MYO15A* are currently identified as loci of autosomal recessive nonsyndromic hearing loss, *DFNB30* and *DFNB3*, respectively (Wang et al., 1998; Walsh et al., 2002). *MYH9* and *MYH14* are identified as chromosomal loci of autosomal dominant nonsyndromic hearing loss, *DFNA17* and *DFNA4A*, respectively (Lalwani et al., 2000; Wang et al., 2000; Donaudy et al., 2004). The other two myosin genes, *MYO6* and *MYO7A*, depending on the variants, are associated with both autosomal dominant and recessive nonsyndromic hearing loss, respectively, as *DFNA22* and *DFNB37* for *MYO6* (Melchionda et al., 2001; Ahmed et al., 2003a) and *DFNA11* and *DFNB2* for *MYO7A* (Liu et al., 1997; Weil et al., 1997). Some variants of *MYO7A* are associated with Usher syndrome type 1. The locus is designated *USH1B* (Weil et al., 1995). Variants of these genes are available in databases such as ClinVar, Deafness Variation Database and gnomAD.

**FIGURE 4:**
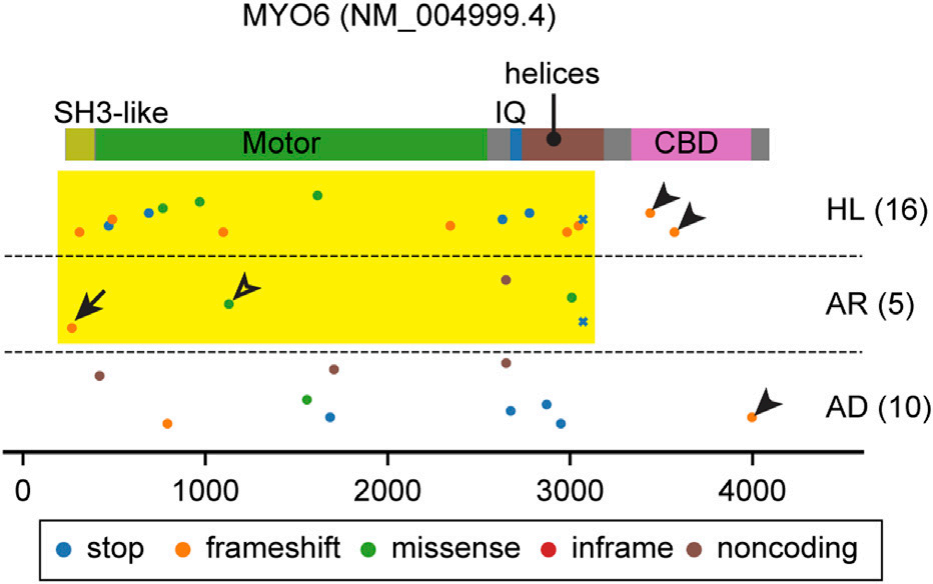
Pathogenic and likely pathogenic MYO6 variants mapped on the domain structure. Most variants affect the motor domain and the neck region (yellow rectangle) except for some frameshift variants in the CBD (arrowheads). See the text for discussion of variants indicated by an arrow (p.Thr13fs) and an open arrowhead (p.Glu299Asp). Variants are obtained and classified as described for Figure 3.

**FIGURE 5:**
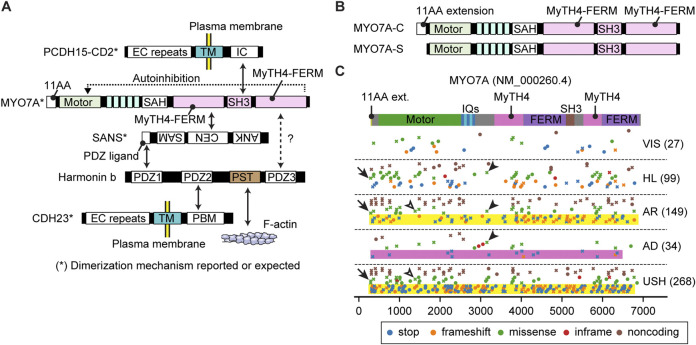
Interactome, N-terminal splicing variations and pathogenic variants of MYO7A **(A)** Interacting partners of MYO7A. SANS and harmonin isoform b (harmonin b) bridge interactions with other partners. Serine and threonine-rich (PST) sequence of harmonin b can bind to F-actin (Grillet et al., 2009). SAH domain of MYO7A has a weak dimerization activity (Sakai et al., 2011; Liu et al., 2021). SANS, PCDH15 and CDH23 can dimerize with each other (Adato et al., 2005; Dionne et al., 2018; Jaiganesh et al., 2018). The tail of MYO7A can inhibit the motor function (autoinhibition). **(B)** Different N-termini of two MYO7A isoforms. The canonical isoform has an eleven amino-acid extension at the N-terminus (MYO7A-C), while the short isoform does not (MYO7A-S). **(C)** Mapping of pathogenic and likely pathogenic MYO7A variants. Obtained and classified as described for Figure 3 adding a category, VIS, to indicate variants associated with retinal dysfunction but not with hearing loss. Nonsense and frameshift variants in *MYO7A* usually result in autosomal recessive Usher syndrome or nonsyndromic hearing loss (yellow rectangles), but some are additionally associated with autosomal dominant nonsyndromic hearing loss (pink rectangle). Overlapping phenotypic categories are observed also for missense mutations (examples shown by open and closed arrowheads). Missense variants are reported for the first methionine codon of the N-terminal extension (p.Met1Val and p. Met1Ile, arrows).

**FIGURE 6:**
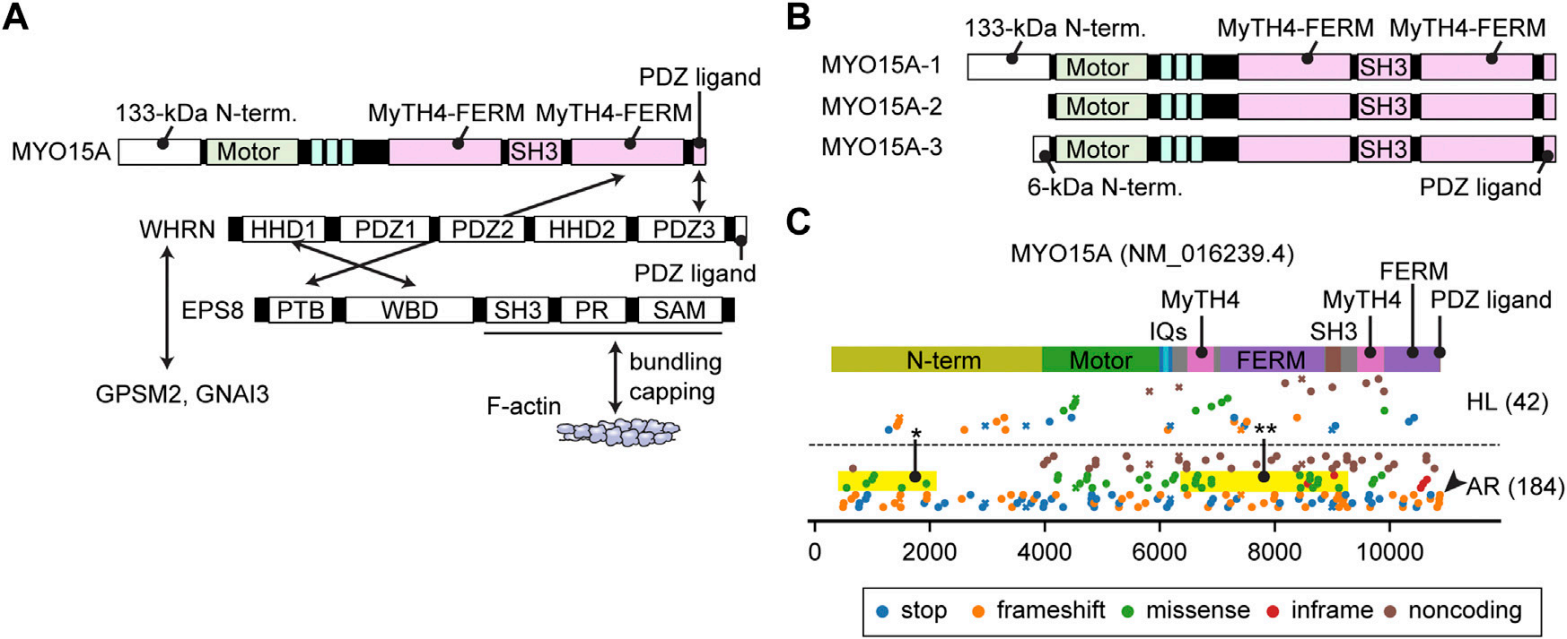
Interactome, N-terminal splicing variations and pathogenic variants of MYO15A **(A)** Known interacting partners of MYO15A. A ternary complex of MYO15A, WHRN and EPS8 interacts with each other. Another ternary of WHRN, GPSM2 and GNAI3 interacts with MYO15A. EPS8 can bundle F-actin and also cap barbed ends. Interacting partners for the first MyTH4-FERM domain and the SH3 domain of MYO15A have not been identified. **(B)** Difference in the N-termini between three MYO15A isoforms. MYO15A-1 has a large 133-kDa N-terminal domain encoded by a single exon, while a short MYO15A-2 isoform does not include this sequence. A novel MYO15A-3 isoform has a small 6-kDa N-terminal extension. **(C)** Mapping of pathogenic and likely pathogenic variants of MYO15A. Variants are obtained and classified as described for Figure 3. Nonsense and frameshift mutations distribute along the entire length of MYO15A. Some missense variants are observed in the N-terminal domain (yellow rectangle with asterisk) and in the MyTH4-FERM and SH3 domains whose binding partners are unknown (yellow rectangle with double asterisk). One variant frameshifts the C-terminal PDZ ligand (arrowhead).

**FIGURE 7:**
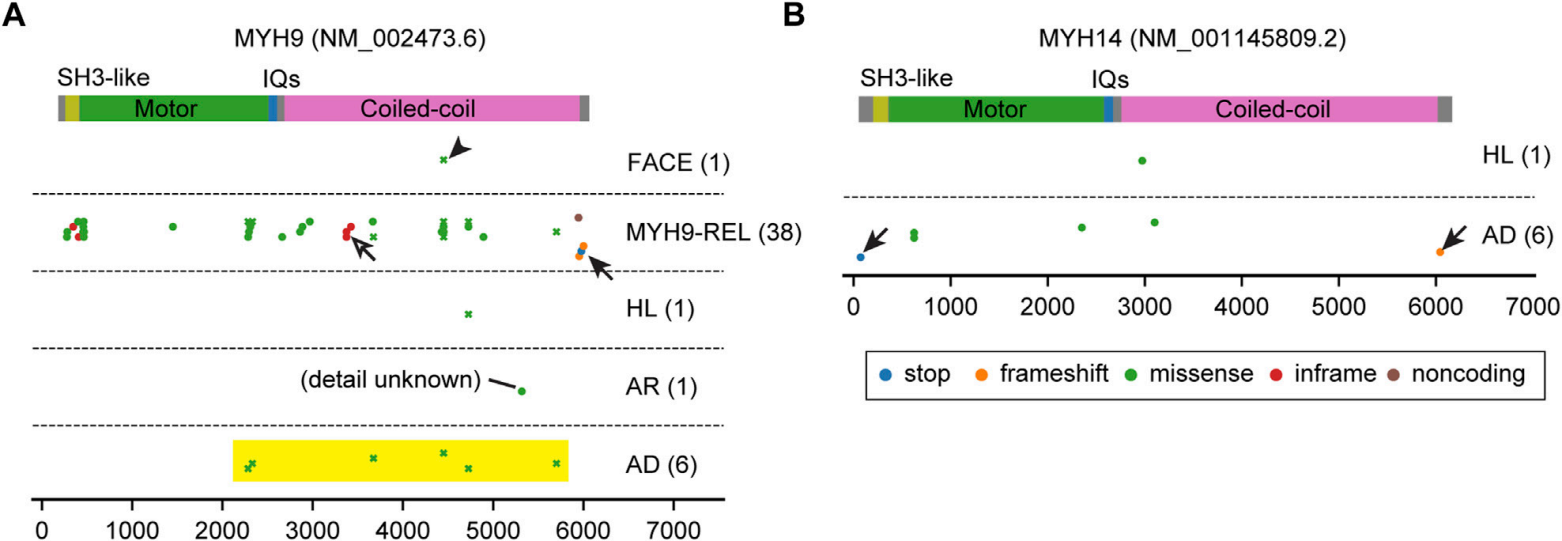
Pathogenic variants of MYH9 and MYH14. **(A, B)** Mapping of pathogenic and likely pathogenic variants in *MYH9* and *MYH14*, correspondingly. Variants are obtained and classified as described for Figure 3 with additional categories for MYH9. Nonsense or frameshift mutations are only found close to the N-terminus or the C-terminus (arrows). Almost all *MYH14* variants are associated with autosomal dominant hearing loss. In contrast, most variants of *MYH9* are associated with *MYH9*-related disorders accompanying platelet disorders and nephritis (MYH9-REL). There is one cluster of inframe mutations (p.Gln1068_Leu1074dup, p. Gln1068_Leu1074del and p. Glu1084del) in the coiled-coil domain (open arrow) for MYH9-REL. One variant is associated with a complex of symptoms including abnormal platelets and facial dysmorphology (p.Asp1424Gly, arrowhead, categorized as FACE). All *MYH9* missense variants associated with autosomal dominant hearing loss occur in the motor domain near the neck or in the neck or tail (yellow rectangle) and are associated also with *MYH9*-related disorders.

Correlation between variants of these myosin genes and clinical phenotypes is crucial for hearing researchers to (1) elucidate the pathophysiology of hearing loss, (2) predict the outcome of a given variant in a patient and (3) formulate therapeutic strategies for hearing loss. For clinicians, it would be best if the audiological prognosis is predictable including thresholds at each frequency in the pure tone audiogram, speech discrimination, time course in loss of hearing ability, mode of inheritance and penetrance. AlphaMissense (Cheng et al., 2023) provides *in silico* predictions for missense variants. It is also imperative to include experimental data on how each variant alters the structure or kinetics of a myosin and results in hearing loss in animal models. However, it is challenging to predict if a variant that slightly modifies the kinetics of a protein (e.g., the ATPase activity of the motor domain) will be pathogenic or not. These changes in kinetics may cause hearing loss later in life or be pathogenic only for a person with other risk alleles in their genetic background. Correlation analyses between variants and clinical outcomes may some day be complemented by aggregating environmental hazards and risks and safeguards in a patient’s genetic background. Correlations between variants of myosin genes and clinical phenotypes have been reported in several studies (Tamagawa et al., 2002; Riazuddin et al., 2008; Rehman et al., 2016; Kabahuma et al., 2021). We have summarized these observations by mapping pathogenic or likely pathogenic variants available in ClinVar to the domain structures of all the myosins currently associated with human hearing loss (Figure 3B; Figure 4; Figure 5C; Figure 6C; Figure 7). In the subsections below, we examine the functions and structures of myosins necessary for normal hearing, raise questions about protein trafficking in stereocilia and further discuss the spectrum of variants in these myosin genes associated with hearing loss.

### 3.2 MYO3A

MYO3A has a 30-kDa kinase domain at the N-terminus and a tail that can interact with MORN4, isoform 1 of ESPN and F-actin (Figure 3A). The N-terminal kinase domain can phosphorylate two residues in the “loop 2” of the motor domain connecting the upper and lower 50-kDa domains and reduce the maximum actin-activated ATPase activity (*k*_cat_) and affinity for F-actin (Quintero et al., 2010). This phosphorylation may auto-regulate the MYO3A motor activity in F-actin protrusions, such as filopodia and stereocilia (Quintero et al., 2010). The kinase domain itself has two threonine residues that can be phosphorylated, of which the C-terminal threonine has a larger impact on the kinase activity (Quintero et al., 2013). Phosphorylation of the C-terminal threonine residue in the kinase domain is mediated by another MYO3A molecule or cellular kinases, likely increasing the activity of the MYO3A kinase domain (Quintero et al., 2013). This myosin was initially considered to have a high duty ratio (∼0.9) with fast phosphate release and slow ADP release (Dose et al., 2007). However, more recent analyses indicate that MYO3A has a low duty ratio (0.25) after removing the kinase domain, which enhances the ATPase activity by two-fold (Dose et al., 2008).

The MYO3A tail domain is partially unfolded but has region/domains that interact with MORN4, THDI (tail homology domain I) and THDII (tail homology domain II). MORN4 can tether its interacting partners to the plasma membrane (Li et al., 2019). THDI and THDII interact with the Ankyrin (ANK) repeat of ESPN isoform 1 or ESPNL and directly with F-actin, respectively (Les Erickson et al., 2003; Salles et al., 2009; Liu et al., 2016). Co-expression of human MYO3A lacking the kinase domain and mouse ESPN isoform 1 fragments in COS-7 cells showed elongated filopodia suggesting that constitutively active MYO3A (without regulation by the kinase domain) may recruit ESPN isoform 1 to stereocilia and boost elongation of F-actin cores (Salles et al., 2009). Concordantly, the ESPN isoform 1 knockout mouse and the jerker mouse show short, thin, immature stereocilia (Sekerkova et al., 2011; Ebrahim et al., 2016). However, a MYO3A without the kinase domain is not reported to be present in stereocilia. How the kinase domain regulates MYO3A function in stereocilia elongation, and if MYO3A with the kinase domain might be a processive motor remain to be elucidated. Interestingly, in mice, the loss of function of MYO3A is likely compensated by MYO3B. *Myo3a* ^−/−^ mice have normal hearing at 1 month of age while a double knockout of *Myo3a* and *Myo3b* results in profound deafness and a dysmorphic staircase architecture of hair cell stereocilia bundles (Lelli et al., 2016).

In humans, variants of *MYO3A* are associated with autosomal recessive nonsyndromic hearing loss, DFNB30 (Walsh et al., 2002). ClinVar aggregates different types of variants in this gene and associates them with autosomal recessive hearing loss or hearing loss with no description of mode of inheritance (AR and HL in Figure 3B). Most variants of *MYO3A* are nonsense mutations, frameshift mutations or mutations in the non-coding region and located in the kinase domain, the motor domain or the neck region (Figure 3B, yellow rectangle). These variants likely disrupt motor function by inserting a premature stop codon or by causing a shift in the reading frame (frameshift) of the mature mRNA. Two frameshift variants are reported in the THDI domain, which may disable the recruitment of ESPN isoform 1 or ESPNL to stereocilia (Figure 3B, arrowheads). Three missense mutations in the kinase domain (p.Leu239Pro) and in the motor domain (p.Gly488Glu and p. Leu697Trp) are each associated with autosomal dominant hearing loss (Grati et al., 2016; Dantas et al., 2018; Doll et al., 2020) (AD in Figure 3B). Although the precise mechanism of the dominant inheritance is unknown, biochemical experiments show that the two mutations in the motor domain lower the ATPase activity and alter its velocity in an F-actin sliding assay (increased by p. Gly488Glu; decreased by p. Leu697Trp) (Grati et al., 2016; Dantas et al., 2018).

A remaining question is how MYO3A traffics in a stereocilium. Currently, there is no evidence that MYO3A has a dimerization sequence, such as a coiled-coil domain, to “walk” on F-actin as a dimer or an oligomer (De La Cruz and Ostap, 2004). The low duty ratio (∼0.25) of the motor domain in the absence of the kinase domain contradicts processive walking (Dose et al., 2008). To move in filopodia and stereocilia, MYO3A requires interaction with F-actin through THDII in order to perhaps cause “inchworm-like” movements (Salles et al., 2009; Raval et al., 2016). ESPN isoform 1 (or ESPNL) may assist MYO3A to move in actin protrusions considering that the tail of MYO3B, which lacks THDII to interact with F-actin, can target filopodia tips only when co-expressed with ESPN isoform 1 (Merritt et al., 2012). Increase of the duty ratio by the kinase domain could be advantageous for MYO3A to move on F-actin using its THDII domain or its cargo, ESPN isoform 1, as a scaffold although the phosphorylated motor domain also decreases its affinity for F-actin (Komaba et al., 2003; Komaba et al., 2010). It is still uncertain how class-III myosins move in F-actin protrusions including stereocilia.

### 3.3 MYO6

All but one myosin studied to date move on F-actin toward the barbed end. The exception is MYO6, which moves toward the pointed end of F-actin. MYO6 has an insertion called the “reverse gear” located between the converter domain and the lever arm (Wells et al., 1999; Shin et al., 2004) that is necessary for movement to the pointed end of F-actin. MYO6 also has a unique tail consisting of a single 26-kDa globular cargo binding domain (CBD) with multiple motifs to interact with a wide variety of binding partners involved in autophagy (TAX1BP, OPTN and NDP52), endocytosis (Dab2, LMTK2, TOM1/L2), mitophagy (Ubiquitin) and G-protein signaling (GIPC) (de Jonge et al., 2019) (Figure 2E). These proteins localize on the cytoplasmic side of vesicles or membrane pits, including those involved in endocytosis, and are considered to tether MYO6A to these structures (Buss and Kendrick-Jones, 2008). It is also known that MYO6 can directly interact with PIP2 in the plasma membrane through its C-terminal lipid-binding motif (Spudich et al., 2007). Some of these interacting partners, such as Dab2, OPTN and GIPC, can dimerize or oligomerize MYO6 (Phichith et al., 2009; Yu et al., 2009; Shang et al., 2017; Chatterjee et al., 2023). Despite these advances in understanding MYO6 function, MYO6 functions in the inner ear, especially in hair cells, is not fully understood. Loss of MYO6 function in Snell’s waltzer mice (*Myo6* ^*sv/sv*^) results in profound hearing loss accompanying fusion of adjacent stereocilia and subsequent degeneration of the organ of Corti (Avraham et al., 1995). Bifurcated stereocilia often observed in this mouse line suggest that MYO6 anchors the plasma membrane to the F-actin core (Self et al., 1999). MYO6 also interacts with tyrosine phosphatase receptor type Q (PTPRQ), a membrane protein crucial to tether the plasma membrane to the F-actin stereocilia core (Avraham et al., 1997; Goodyear et al., 2003; Sakaguchi et al., 2008). Furthermore, it is uncertain if MYO6 loaded with cargo traffics in a stereocilium. MYO6 ceases stepping and behaves as a molecular anchor rather than a molecular transporter when (1) under load and (2) at subsaturated ATP or in the presence of ADP (Altman et al., 2004). MYO6 may anchor stereocilia rootlets to the hair cell apical plasma membrane through PTPRQ, exerting force to help keep each stereocilium in place (Cramer, 2000).

Both autosomal dominant DFNA22 and recessive nonsyndromic hearing loss DFNB37 deafness are associated with variants of *MYO6* (Melchionda et al., 2001; Ahmed et al., 2003a). Most variants of MYO6 affect the motor domain and the neck region (Figure 4, yellow rectangle) except for some frameshift variants in the CBD (Figure 4, arrowheads). Unfortunately, many variants of *MYO6* are reported in ClinVar without information about the mode of inheritance (Figure 4). However, heterozygous *Myo6* ^*+/sv*^ and *Myo6* ^+/−^ mice suggest that haploinsufficiency of MYO6 function in the inner ear explains the progressive hearing loss (Karolyi et al., 2003; Seki et al., 2021). A frameshift mutation at the N-terminus (p.Thr13fs), located in annotated exon 1 of *MYO6*, is associated with autosomal recessive hearing loss, *DFNB37* (Figure 4, arrow). For this variant, perhaps the MYO6 transcript may be translated from the methionine codon at nucleotide position 18 (NM016239.4) or further downstream. A second possibility is alternative splicing that includes a “hidden” 1^st^ exon located in highly conserved sequence in intron 1 but which in a homozygous p. Thr13fs individual is insufficient in producing enough functional MYO6 for a normal phenotype. A missense variant (p.Glu299Asp) in the motor domain is associated with autosomal recessive hearing loss, which may be the result of a slightly hypofunctional motor domain whose activity is insufficient when homozygous but sufficient when heterozygous (Figure 4, open arrowhead).

### 3.4 MYO7A

MYO7A has a long tail domain of approximately 1,200 residues consisting of two MyTH4-FERM domains and one SH3 domain (Figure 2E). MYO7A localizes at the upper tip-link density (UTLD) with other components of the tip-link complex, USH1C (harmonin), SANS and CDH23 (Grati and Kachar, 2011), and tethers PCDH15 at the tip of an adjacent shorter stereocilium (Kazmierczak et al., 2007) (Figure 1D). PCDH15 is mechanically connected to the MET channel complex composed of TMC1/TMC2 and accessory proteins, TMIE, CIB2 and probably LOXHD1 (Naz et al., 2002; Zhao et al., 2014; Kurima et al., 2015; Giese et al., 2017; Trouillet et al., 2021). Genes encoding the components of the tip-link complex and the MET channel complex, *USH1C*, *CDH23*, *PCDH15*, *TMIE* and *CIB2*, are associated with autosomal recessive nonsyndromic hearing loss, DFNB18, DFNB12, DFNB23, DFNB6 and DFNB48, respectively (Bork et al., 2001; Naz et al., 2002; Ahmed et al., 2003b; Riazuddin et al., 2012). Some components of the tip-link complex are also utilized in photoreceptors (Cosgrove and Zallocchi, 2014). In addition to MYO7A, four genes encoding the tip-link complex, *USH1C*, *SANS*, *CDH23* and *PCDH15*, are associated with Usher syndrome type 1 (Verpy et al., 2000; Ahmed et al., 2001; Alagramam et al., 2001; Bolz et al., 2001; Weil et al., 2003). MYO7A is also involved in transporting twinfilin-2 to stereocilia tips (Rzadzinska et al., 2009) and formation of the ankle link through interaction with a scaffolding protein PDZD7 (Grati et al., 2012; Morgan et al., 2016).

MYO7A and their interacting partners are essential components of the tip-link complex. Recent studies have elucidated an interactome among MYO7A and cargo (Figure 5A). Mouse models disabling or partially disabling the tip-link components have been useful tools to understand the formation of the tip-link complex. For example, localization of harmonin B at the UTLD is lost in mice with defective MYO7A (*Myo7a* ^*4626SB/4626SB*^) or SANS (*Ush1g* ^*js/js*^) but retained in mice with mutant CDH23 (*Cdh23* ^*v2J/v2J*^) or PCDH15 (*Pcdh15* ^*av3J/av3J*^) (Lefevre et al., 2008) suggesting that localization of harmonin B is dependent on MYO7A and SANS. The F-actin binding motif of harmonin B is essential for forming the UTLD and anchoring the tip-link to the F-actin core but not necessary for transporting the tip-link complex and harmonin B itself (Grillet et al., 2009). The eleven amino-acid extension of the canonical isoform of MYO7A (MYO7A-C in Figure 5B) may be crucial for maintaining the tip-link complex because mice lacking the MYO7A-C isoform show reduced resting open probability and slowed onset of MET currents and progressive hearing loss (Li S. et al., 2020). The tip-link components are likely replenished continuously because postnatal deletion of *Ush1c* and *Ush1g* alleles show progressive loss of tip-links and the corresponding mutant mice are profoundly deaf (Caberlotto et al., 2011). Interestingly, postnatal deletion of *Myo7a* shows progressive hearing loss and results in profound deafness in mice around P60 accompanying a decrease in MET currents without the loss of tip-links (Corns et al., 2018). Once tip-links are formed, the motor activity of MYO7A is probably not required to replenish their components.

Compared with its binding partners, less is known about how the motor activity of MYO7A is utilized in a stereocilium. Proteins in the interactome of MYO7A may provide several mechanisms for MYO7A to move in a stereocilium, (1) dimerization, (2) anchoring to the plasma membrane via CDH23 or PCDH15 (Dionne et al., 2018; Jaiganesh et al., 2018) and (3) binding to F-actin via harmonin B (Grillet et al., 2009) (Figure 5A). For dimerization, the SAH domain of MYO7A likely has a weak dimerization activity considering that a small portion of *Drosophila* myosin VIIa fragment (head + neck) can spontaneously dimerize and walk on F-actin (Liu et al., 2021) and that the SAH domain contains a motif resembling a leucine zipper (Sakai et al., 2011). In addition, SANS, PCDH15 and CDH23 can dimerize with each other and may keep multiple MYO7A molecules in close proximity (Adato et al., 2005; Dionne et al., 2018; Jaiganesh et al., 2018). Two other mechanisms may be utilized by MYO10 and MYO3A to move in F-actin protrusions. Anchoring to the plasma membrane can be used by MYO10 to form F-actin protrusions at the cell edge and then move toward the tip in the protrusion (Fitz et al., 2023). Binding to F-actin is necessary for MYO3A to move in a stereocilium or a filopodium (Salles et al., 2009; Raval et al., 2016). An open question is why MYO7A shows slow movements as a dimer, especially when compared with MYO10, which is a motor with high duty ratio functioning in filopodia (Takagi et al., 2014). MYO7A and MYO10 show similar velocities in the F-actin sliding assay, 0.19 μm/s and 0.17 μm/s, respectively (Homma and Ikebe, 2005; Watanabe et al., 2006). However, these two myosins show different kinetics in single-molecule microscopy experiments using homodimers. Dimerized human MYO7A can only move at 11.0 ± 0.6 nm/s (Sato et al., 2017), while dimerized MYO10 can move at 578 ± 174 nm/s in filopodia (Kerber et al., 2009). Interestingly, movements of human MYO7A are slower than *Drosophila* myosin VIIa (crinkled), 72 ± 20 nm/s (Sato et al., 2017). The slow movements of MYO7A might prevent this motor protein from behaving like MYO10, which makes cellular protrusions when anchored to the plasma membrane (Fitz et al., 2023).

Variants of MYO7A are associated with hearing loss and/or retinal pathology (Figure 5C). Nonsense and frameshift variants in *MYO7A* usually result in autosomal recessive Usher syndrome type 1 or nonsyndromic hearing loss (yellow rectangles, Figure 5C). However, some nonsense and frameshift variants are also associated with autosomal dominant nonsyndromic hearing loss (pink rectangle, Figure 5C, plotted by crosses to indicate association with multiple phenotypes). Similar overlaps are observed in missense mutations (open and closed arrowheads, Figure 5C). Furthermore, 27 variants can cause only retinal symptoms (VIS in Figure 5C). Variable expressivity of *MYO7A*, and perhaps genes related to *MYO7A*, may be a reason why one variant can show different phenotypes (Zina et al., 2001). Correlation between genotypes and phenotypes, such as onset and severity of hearing loss and presence of retinal dysfunction, is complex. Currently, it is still challenging to correlate genotypes and phenotypes in Usher syndrome (Galbis-Martinez et al., 2021). Missense variants are reported in the first methionine of the N-terminal extension (p.Met1Val and p. Met1Ile) and are associated with Usher syndrome and autosomal recessive hearing loss (arrows in Figure 5C) suggesting the importance of this extension in human for intact functions of the retina and stereocilia.

### 3.5 MYO15A

The domain structure of the MYO15A tail is similar to MYO7A. Both have two MyTH4-FERM domains and one SH3 domain (Figure 2E). One large difference from MYO7A is a 133-kDa N-terminal domain encoded by exon 2 of MYO15A (Figure 6B). Formerly, two isoforms with and without this N-terminal extension were found and referred to as MYO15A-L and MYO15A-S. Recently, a third isoform with a small 6-kDa N-terminal extension was identified in cochlear hair cells (Rehman et al., 2016; Ranum et al., 2019) renaming the three isoforms as MYO15A-1, MYO15A-2 and MYO15A-3, respectively (Figure 6B). MYO15A-1 is localized at the tips of short stereocilia near PCDH15 and is essential for maintaining the length of the mechanotransducing stereocilia (Fang et al., 2015). MYO15A-2 lacking the N-terminal domain is crucial for elongating stereocilia to a developmentally predetermined length and can rescue abnormally short stereocilia in *Shaker-2* mice (Belyantseva et al., 2003). Although the exact function of MYO15A-3 is unknown, a previous review points to its involvement in trafficking of BAIAP2L2 (Moreland and Bird, 2022), which is an inverse BAR (I-BAR) domain proteins and interacts with phosphoinositide-rich membrane to induce plasma membrane protrusions (Zhao et al., 2011). The C-terminal PDZ ligand of MYO15A interacts with a scaffolding protein whirlin (Belyantseva et al., 2005), and MyTH4-FERM domain with an F-actin interacting protein EPS8 (Manor et al., 2011) (Figure 6A). The ternary complex of MYO15A, whirlin (WHRN) and EPS8 is essential for elongating the F-actin core. The *shaker-2*, *whirler* and *Eps8* ^*−/−*^ mice fail to elongate stereocilia to a wild-type length (Beyer et al., 2000; Mogensen et al., 2007; Zampini et al., 2011). MYO15A also has the ability to nucleate F-actin, which may contribute to the elongation of the F-actin core (Moreland et al., 2021). The ternary complex of WHRN, GPSM2 and GNAI3 is also part of the MYO15A interactome. WHRN specifies the tallest stereocilia and defines hair bundle row identity (Tadenev et al., 2019). It is unknown how MYO15A traffics in a stereocilia, but the relatively high duty ratio of its motor domain (∼0.5) is consistent with processive movements as a dimer or an oligomer (Jiang et al., 2020).

Currently, variants of *MYO15A* are associated with autosomal recessive nonsyndromic hearing loss DFNB3 (Figure 6C). In addition to the nonsense and frameshift mutations distributed along the entire length of MYO15A and missense mutations in the motor domain, some missense variants are observed in the large N-terminal domain indicating the importance of this sequence (yellow rectangle with asterisk, Figure 6C). One variant truncates the C-terminal PDZ ligand as previously reported (Rehman et al., 2016) (arrowhead, Figure 6C). Interestingly, some missense mutations are reported in the first MyTH4-FERM domain and in the SH3 domain suggesting that these tail domains are sites of yet to be identified binding partners (yellow rectangle with double asterisks, Figure 6C).

### 3.6 Non-muscle class II myosins

Currently, variants of *MYH9* and *MYH14* genes are associated with autosomal dominant nonsyndromic hearing loss, *DFNA17* and *DFNA4A*, respectively (Lalwani et al., 2000; Wang et al., 2000; Donaudy et al., 2004). Class-II myosins contain three non-muscle myosins, NMIIA (MYH9), NMIIB (MYH10) and NMIIC (MYH14) (Heissler and Manstein, 2013). Non-muscle class-II myosins are present in every cell type (Costa and Sousa, 2020). They form contractile actomyosin structures essential for cellular organization, polarity and regulation (Vicente-Manzanares et al., 2009) (Figure 2D). Expression of non-muscle myosins are different among cell types. NMIIA and NMIIB are expressed in endothelial and epithelial cells at similar levels while NMIIB and NMIIC are abundant in nervous and lung tissue, respectively (Kawamoto and Adelstein, 1991; Golomb et al., 2004). NMIIA, NMIIB and NMIIC are expressed in mammalian cochlear epithelia and regulate extension, growth and patterning of the cochlear duct (Yamamoto et al., 2009).

The distribution of *MYH9* and *MYH14* variants are unique compared with variants of unconventional myosins (Figure 7). Nonsense or frameshift variants are only found close to the N-terminus or the C-terminus probably suggesting a dominant negative effect of truncated proteins (arrows in Figure 7). While almost all *MYH14* variants are associated with autosomal dominant hearing loss, most variants of *MYH9* are associated with other *MYH9*-related disorders including platelet disorders and nephritis (Althaus and Greinacher, 2009) (MYH9-REL in Figure 7A). Among the variants associated with *MYH9*-related disorders, there is one cluster of inframe mutations (p.Gln1068_Leu1074dup, p. Gln1068_Leu1074del and p. Glu1084del) in the coiled-coil domain that probably does not have a destructive effect on protein folding (open arrow in Figure 7A). One variant is associated with a pleiotropic phenotype including an abnormality of platelets and facial dysmorphology (p.Asp1424Gly, arrowhead in Figure 7A). All missense variants associated with autosomal dominant hearing loss occur in the motor domain close to the neck, in the neck or in the tail and are also associated with *MYH9*-related disorders (yellow rectangle in Figure 7A and plotted by crosses to indicate association with multiple phenotypes). In patients with these variants, platelet disorders might be overlooked because of (1) bleeding is more frequently associated with variants in the motor domain than variants in the neck and the tail and (2) a tendency toward bleeding (e.g., easy bruising, epistaxis and gum bleeding) can manifest differently even between a parent and a child harboring the same variant of *MYH9* (Saposnik et al., 2014).

### 3.7 Disputed or refuted “deafness genes”: *MYO1A*, *MYO1C* and *MYO1F*

To date, three genes encoding the class-I myosins, *MYO1A*, *MYO1C* and *MYO1F*, were initially reported as human “deafness genes” (Donaudy et al., 2003; Zadro et al., 2009). Association of variants of *MYO1A* with deafness was refuted as either the variants of *MYO1A* were identified in healthy controls or the deafness was explained by convincing variants of different genes (Eisenberger et al., 2014; Patton et al., 2016). Absence of any overt pathology in homozygous *Myo1a* knockout mice supports these findings (Tyska et al., 2005). Association of *MYO1C* and *MYO1F* variants with deafness was disputed by the ClinGen Hearing Loss Clinical Domain Working Group (CDWG) due to a lack of sufficient functional analyses (DiStefano et al., 2019). This class of myosins have a neck containing three IQ motifs and a tail with a tail homology 1 (TH1) domain (MYO1A–D, G and H) or two tail homology domains (TH1 and TH2) and one SH3 domain (MYO1E and F) (Diaz-Valencia et al., 2022) (Figure 2E). The TH1 domain has a putative pleckstrin homology (PH) motif and can interact with PIP2 in the lipid bilayer (Hokanson et al., 2006). In the inner ear, MYO1C was reported to be involved in adaptation of MET channels (i.e., rapid closing of activated MET channels) through interaction with the plasma membrane (Stauffer et al., 2005). Future studies may find additional association of class-I myosins with inner ear function.

## 4 Discussion

Genetic studies have identified more than one hundred genes with variants associated with hearing loss including four genes encoding unconventional myosins, *MYO3A*, *MYO6*, *MYO7A* and *MYO15A*, and two genes encoding conventional myosins, *MYH9* and *MYH14*. The four unconventional myosins and some class-I myosins are expressed in hair cells and are essential for developing and maintaining functional stereocilia. The tail domains of these unconventional myosins have motifs unique for each protein and have evolved to interact with specific protein partners and phospholipids (PIP2), which are often referred to as “cargo” transported and/or anchored on the F-actin core of a stereocilium. Major functions of cargo proteins can be largely categorized into (1) formation of the MET machinery, (2) elongation of the F-actin core and (3) tethering the plasma membrane and certain links to the F-actin core (Figure 1D) but not limited to these. For example, Twinfilin-2, which caps F-actin of stereocilia in shorter rows, is identified as cargo of MYO7A (Rzadzinska et al., 2009). In addition to elucidating the functions of cargo proteins in stereocilia development, recent topics include novel isoforms of MYO7A and MYO15A derived from previously “hidden” alternatively spliced small exons (Rehman et al., 2016; Ranum et al., 2019; Li S. et al., 2020).

Compared with the interacting partners of these myosins, less is understood about how the motor activities of these myosins are utilized and regulated in a stereocilium. For example, it is unknown how MYO7A becomes localized at the UTLD and whether or not it is dimerized. In the retina, MYO7A can be recruited to melanosomes by MYRIP which activates the MYO7A motor function through cargo-mediated dimerization (El-Amraoui et al., 2002; Sakai et al., 2011). However, there is no evidence that MYRIP is utilized in hair cell stereocilia although it is present. RNAseq in FACS-sorted hair cells shows higher expression of MYRIP in supporting cells than in hair cells (https://shield.hms.harvard.edu/viewgene.html?gene=Myrip). One scenario is that proteins in the MYO7A interactome provide mechanisms for MYO7A to move in a stereocilium as discussed in the previous section. Another possibility is that MYO7A is directly recruited to the UTLD by the proteins already there, such as CDH23, harmonin B and SANS, although another mechanism would be necessary to recruit these proteins to the UTLD in this scenario. A similar challenge can be posed to other myosins, such as MYO6 and MYO15A, for which the entire interactome in hair cells needs to be clarified. Utilization of motor activities in stereocilia may be difficult to analyze using conventional mouse models lacking functional myosins or their cargo because stereocilia in these mouse models are often severely deformed, disturbing the cargo transport system in them (Lefevre et al., 2008). To approach utilization and regulation of motor activities in a stereocilium, advances in single molecule visualization methodologies will be required to observe the *in vivo* behaviors of myosins and cargo in real time in live hair cell stereocilia.

In addition, we speculate that more detailed clinical information of patients is necessary for correlation analyses between variants and phenotypes. Large databases, such as ClinVar and gnomAD, aggregate variants with clinical symptoms. However, even from these carefully curated databases, it is difficult to obtain detailed measurements necessary for hearing research, such as thresholds at each frequency in the pure tone audiograms, speech discrimination, time course in loss of hearing ability and the clinical conditions of patients including accompanying symptoms, because these details are written in a format different for each journal, database and website. From a technical point of view, more flexible approaches, such as artificial intelligence and machine learning, may be useful to handle large and often unformatted data. Artificial intelligence has already been introduced into clinical genomics laboratories and utilized to predict the pathogenicity and phenotypic consequence of variants (Aradhya et al., 2023). With the ability to handle large data, these novel tools may assist hearing researchers to discover correlations between variants and phenotypes and to identify variants of high priority.

Active transport by myosins are crucial for developing cell protrusions including stereocilia, microvilli and filopodia (Houdusse and Titus, 2021). Elucidating the pathophysiology of hearing loss caused by myosin variants will be useful to formulate therapeutic strategies not only for sensorineural hearing loss but also for other diseases such as cancer, virus infection and inflammatory bowel diseases.

## Abbreviations

ATP, adenosine triphosphate; ADP, adenosine diphosphate; PIP2, phosphatidylinositol-4,5-bisphosphate; *DFNA*, chromosomal locus for dominantly inherited nonsyndromic deafness; *DFNB*, chromosomal locus for recessively inherited nonsyndromic deafness; ELC, essential light chain; RLC, regulatory light chain; CaM, calmodulin; IQ-motif, isoleucine-glutamine-rich motif, USH, Usher syndrome; OHC, outer hair cell; IHC, inner hair cell; RT, room temperature.
